# Qualitative speed-accuracy tradeoff effects can be explained by a diffusion/fast-guess mixture model

**DOI:** 10.1038/s41598-021-94451-7

**Published:** 2021-07-26

**Authors:** Roger Ratcliff, Inhan Kang

**Affiliations:** The Ohio State University, 1835 Neil Avenue, Columbus, OH 43210 USA

**Keywords:** Psychology, Human behaviour, Computational models

## Abstract

Rafiei and Rahnev (2021) presented an analysis of an experiment in which they manipulated speed-accuracy stress and stimulus contrast in an orientation discrimination task. They argued that the standard diffusion model could not account for the patterns of data their experiment produced. However, their experiment encouraged and produced fast guesses in the higher speed-stress conditions. These fast guesses are responses with chance accuracy and response times (RTs) less than 300 ms. We developed a simple mixture model in which fast guesses were represented by a simple normal distribution with fixed mean and standard deviation and other responses by the standard diffusion process. The model fit the whole pattern of accuracy and RTs as a function of speed/accuracy stress and stimulus contrast, including the sometimes bimodal shapes of RT distributions. In the model, speed-accuracy stress affected some model parameters while stimulus contrast affected a different one showing selective influence. Rafiei and Rahnev’s failure to fit the diffusion model was the result of driving subjects to fast guess in their experiment.

## Introduction

Would we want to apply a single-process model to a task in which on some proportion of the trials subjects try to make a decision about a stimulus while on other trials they close their eyes and hit a button randomly? Rafiei and Rahnev^1^ presented an experiment in which subjects were asked to judge the orientation of a Gabor patch with five levels of contrast. They used five levels of speed-stress, varying from extremely high stress on speed to high stress on accuracy (“slow” stress in their terms). Our analyses of their data show that when speed was stressed, on some proportion of trials subjects randomly responded to the onset of the stimulus without waiting to make a decision based on stimulus orientation. The results produced bimodal response time (RT) distributions in the speed-stress conditions for some subjects and distributions with most RTs less than 300 ms for others. The accuracy of the responses with RTs less than 300 ms was chance.

**Figure 1:**
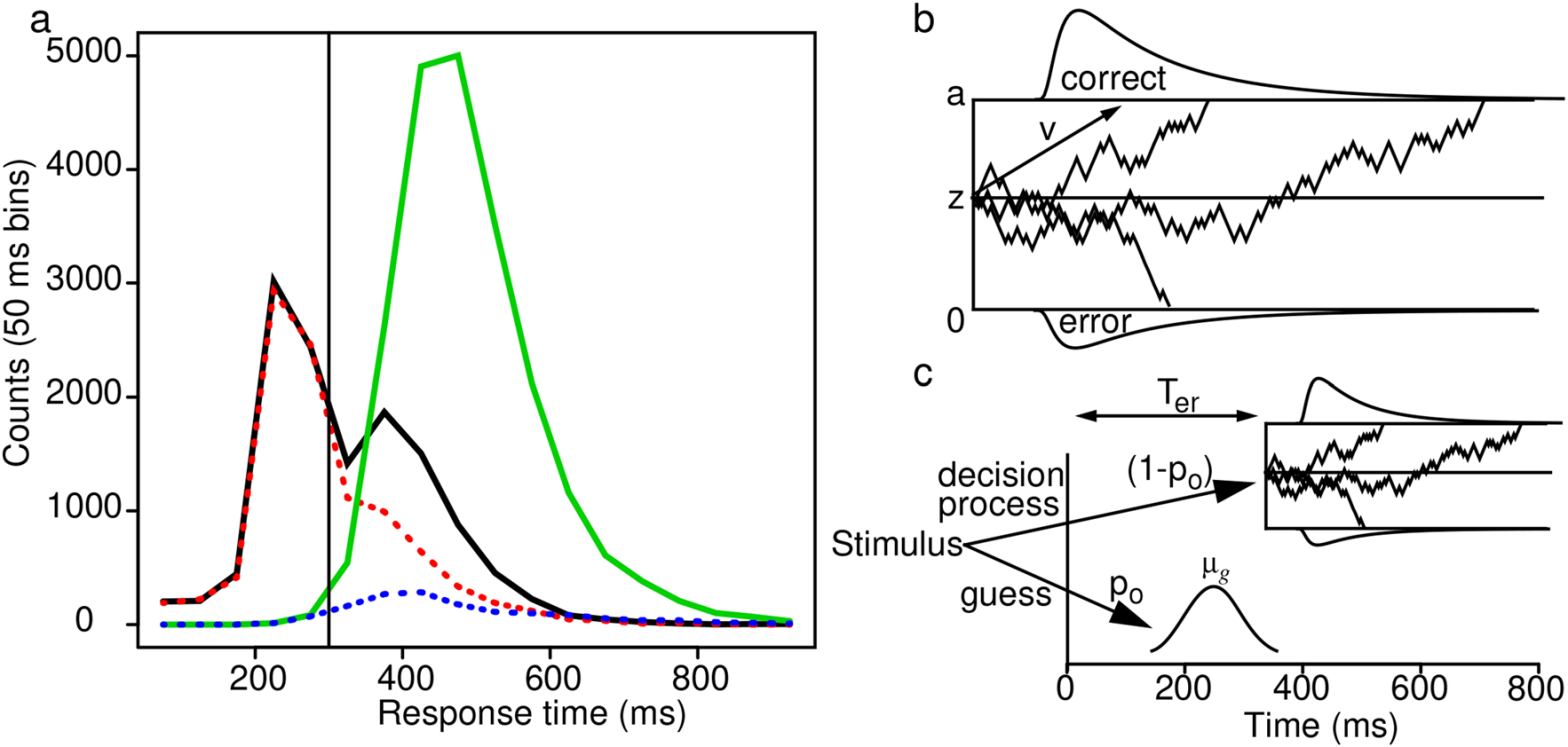
(**a**) The black solid line and red dashed line show distributions of RTs collapsed over conditions with the lowest accuracy. These combined the 5 stimulus contrast conditions with the highest speed-stress plus the lowest stimulus contrast condition with the second to highest speed-stress. The black solid line is for correct responses and the red dashed line is for errors. The green solid line and blue dotted line show distributions for the conditions with highest accuracy. These are the three stimulus contrast conditions with highest discriminability for the slowest speed-accuracy condition and the two conditions with the highest discriminability for the second slowest condition. The green solid line is for correct responses and the blue dotted line is for errors. The vertical line is at 300 ms and the parts of the RT distributions below it completely overlap with chance accuracy. The distributions in (**a**) are wider than would be representative of any individual because all responses from all subjects are included in the analysis (Fig. 3 shows narrower distributions for individual subjects, see the discussion in Ratcliff^12^, p. 454). (**b**) An illustration of the diffusion model. (**c**) An illustration of the mixture model showing the probabilities of guesses (*p*_*o*_) and diffusion processes (1-*p*_*o*_). The relative onset times for the two processes are also shown.

To demonstrate this, Fig. 1a shows correct and error RT distributions from Rafiei and Rahnev’s data for conditions with highest speed-stress and lowest accuracy and for conditions with lowest speed-stress and highest accuracy (in each case, all RTs are grouped over subjects and conditions). The figure shows that the correct and error RT distributions completely overlap for RTs less than 300 ms and the bimodal distribution for correct responses diverges from the distribution for error responses only for RTs greater than 300 ms. In their analyses, Rafiei and Rahnev used a cutoff of 150 ms, but Fig. 1a shows accuracy starts to rise above chance only after about 300 ms.

Rafiei and Rahnev’s research represents a rediscovery of fast guessing. Fast guessing was a major topic in the 1960’s and 1970’s with experiments with simple stimuli that examined the phenomenon and models that were developed to account for the data (e.g.^2–4^). Luce^5^ reviewed fast guess data and modeling from a range of experimental tasks including those from a study by Blough^6^ with pigeons performing a (light) wavelength discrimination task and a study by Swensson^7^ with humans performing a discrimination task judging the orientation of a rectangle. In both cases, the shape of RT distributions with RTs less than about 250 ms was unaffected by manipulations of difficulty. Discriminative responses had RTs greater than about 300 ms in Swensson’s study and in Blough’s study, and there were peaks in the RT distributions at 200 ms for fast guesses and at 350 ms for discriminative responses based on the stimulus.

Rafiei and Rahnev’s article focussed on showing that the standard diffusion model^8,9^ cannot account for the results from their experiment. In the model (Fig. 1b), information is accumulated from a starting point, *z*, until the process reaches one of the two response criteria, or boundaries, *a* and *0*. The drift rate of the accumulation process, *v*, is determined by the quality of the stimulus information. Drift rate is assumed to vary (from a normal distribution) from trial to trial with SD η and the starting point of the process is also assumed to vary from trial to trial with range *s*_*z*_. The time for processes outside the decision process (encoding, transformation of the stimulus representation to the decision-relevant representation, and response output) is assumed to have mean *T*_*er*_ and range *s*_*t*_. (Ratcliff^8,10^ showed that the precise forms of the across-trial distributions are not critical).

Fast guesses have been considered in some applications since the research described in Luce’s review. Ratcliff^11^ examined the effects of fast and slow outlier RTs on the power of analysis of variance and on measures such as skewness and kurtosis^12^. Ratcliff and Tuerlinckx^13^ discussed fast guesses in the context of parameter estimation for the diffusion model. The DMAT diffusion model fitting package^14^ has an explicit mechanism to detect the point in time at which accuracy begins to rise above chance which can be used as a cutoff to eliminate fast guesses. However, this outlier-elimination mechanism has not found much use as far as we know. Simen et al.^15^ conducted an optimality analysis of the diffusion model and found that in some cases in which one stimulus occurred with greater probability than the other, fast guesses were optimal to maximize reward rate. In these studies and others, it is generally agreed that if subjects are encouraged to respond extremely quickly, some subjects some proportion of the time will engage in fast guessing. Generally, a lot of diffusion modeling has used cutoffs (i.e., RTs below which accuracy is at chance) to eliminate fast guesses or has eliminated subjects with a large proportion of fast guesses.

In many experiments, fast guessing has been minimized by instructions, monitoring subjects, and/or an extra long delay following a too-fast response to discourage such responses (i.e., a time penalty). Remaining too-fast responses have usually been eliminated from analyses and model fitting. However, recently, experiments are being published with results contaminated with fast guesses, and online tasks or those without close supervision of subjects are susceptible to fast guesses^16,17^.

## Methods and results

To directly address Rafiei and Rahnev’s analyses of their data and the problems with them, we introduce a mixture model that adds a simple, explicit model of fast guessing to the standard diffusion process for stimulus-based discriminative decisions. In this model, there is some probability at each level of speed-stress that a trial is a fast guess. Figure 1c shows the model with a probability *p*_*o*_ of a fast guess and a probability *1* − *p*_*o*_ of a standard diffusion process. *p*_*o*_ differs as a function of the speed-accuracy condition, but is constant across levels of stimulus contrast. Fast guesses are assumed to be random with 50% accuracy and guessing times are normally distributed with mean m_*g*_ and standard deviation (SD) *s*_*g*_ that are fixed across all conditions. While the normal distribution is likely an oversimplification, a slightly skewed distribution such as an inverse Gaussian (e.g.^8,11,18,19^) would provide similar results at the expense of an additional parameter.

The mixture model was fit to Rafiei and Rahnev’s data for each subject separately with all of its parameters estimated simultaneously for all of the experimental conditions. There were 25 conditions, five levels of speed-stress crossed with five levels of stimulus contrast. There were 25 parameters: 5 each for boundary separation, nondecision time, drift rates, and the probability of a fast guess. The mean and SD of the normal distribution for guesses were held constant across all conditions. There were also 3 across-trial variability parameters, also held constant across all conditions: the SD in drift rate, the range in nondecision time, and the range in starting point (which is equivalent to across-trial variability in boundary separation when it is less than half the boundary separation). This 25-parameter model had to fit correct and error RT distributions and accuracy, simultaneously, for all 25 conditions. To anticipate, the model fit the data well with selective influence, something that Rafiei and Rahnev claimed the model could not do. Drift rate changed only across stimulus contrast conditions and not speed-accuracy conditions, and boundary separation, nondecision time, and the probability of fast guesses changed only across speed-accuracy conditions and not stimulus contrast conditions.

The model was fit using 9 quantile RTs, the 0.1, 0.2, 0.3,…, and 0.9 quantiles^20^. The quantile RTs and parameter values of the model were used to generate the predicted cumulative probabilities of a response by that quantile RT. Subtracting the cumulative probabilities for each successive quantile from the next higher quantile gives the expected proportion of responses between adjacent quantiles (π_i_). The observed proportion of responses between adjacent quantiles is *p*_*i*_ = 0.1. The model was fit by minimizing the G-square multinomial maximum likelihood statistic *G*^*2*^ = *2* Σ *N p*_*i*_* ln(p*_*i*_*/*π_i_), where *N* is the number of observations for the condition. This statistic is equal to twice the difference between the maximum possible log likelihood and the log likelihood predicted by the model (because *ln(p/*π) = *ln(p)* − *ln(*π)).

With 9 quantile RTs to represent correct and error RT distributions for the 25 conditions^20^, there were 10 degrees of freedom for each distribution because there are 10 bins outside and between the 0.1, 0.2, 0.3, …, 0.9 RT quantiles for both correct and error distributions. The total probabilities for each pair of correct and error RTs must add to 1, so the number of degrees of freedom for each condition, with correct and error RT distributions, is 19 (=20–1) giving 475 degrees of freedom to be explained by the 25 parameters of the model. There were two modifications for fitting the data. One was that we set the probability of fast guesses to zero if the proportion was less than 0.1. When the estimated proportion of fast guesses was so small, then for conditions with small error rates, there were sometimes mispredictions because these error conditions were not influential in determining the proportion of fast guesses. Setting the proportion to zero corrects this bias. The other modification was that the values of boundary separation and nondecision time were set equal for the two highest speed-stress conditions. This was done because for some of the subjects, the probability of fast guesses was very high (over 0.98) for the highest speed-stress condition which means that these parameters were identified poorly because of the small numbers of error responses (2%). The fits with parameters in Table 1 are from means across the parameters from fits of the individual subjects.

**Table 1:** Diffusion model parameters for the guessing model.

*a*_*1*_	*a*_*2*_	*a*_*3*_	*a*_*4*_	*a*_*5*_	*T*_*er1*_	*T*_*er2*_	*T*_*er3*_	*T*_*er4*_	*T*_*er5*_	*p*_*o1*_	*p*_*o2*_	*p*_*o3*_	*p*_*o4*_	*p*_*o5*_
0.063	0.063	0.072	0.083	0.106	0.356	0.356	0.382	0.393	0.414	0.735	0.201	0.0	0.0	0.0

*v*_*1*_	*v*_*2*_	*v*_*3*_	*v*_*4*_	*v*_*5*_	η	*s*_*z*_	*s*_*t*_	μ_*g*_	*s*_*g*_					
0.108	0.175	0.283	0.426	0.546	0.184	0.037	0.129	0.262	0.051					

The numerical index for boundary separation (*a*), nondecision time (*T*_*er*_), and fast guess proportions (*p*_*o*_) represent speed accuracy conditions, 1 for extremely fast, 2 for fast, 3 for medium, 4 for slow, and 5 for extremely slow. The numerical index for drift rate (*v*) represents the contrast (difficulty) manipulation, 1 for the lowest contrast and 5 for the highest. The SD in drift across trials is η, the range of the distribution of starting point is *s*_*z*_, the range of the distribution of nondecision times is *s*_*t*_, the mean of the normal distribution of guesses is μ_*g*_ and the SD in the distribution of guesses is *s*_*g*_*.*

**Figure 2:**
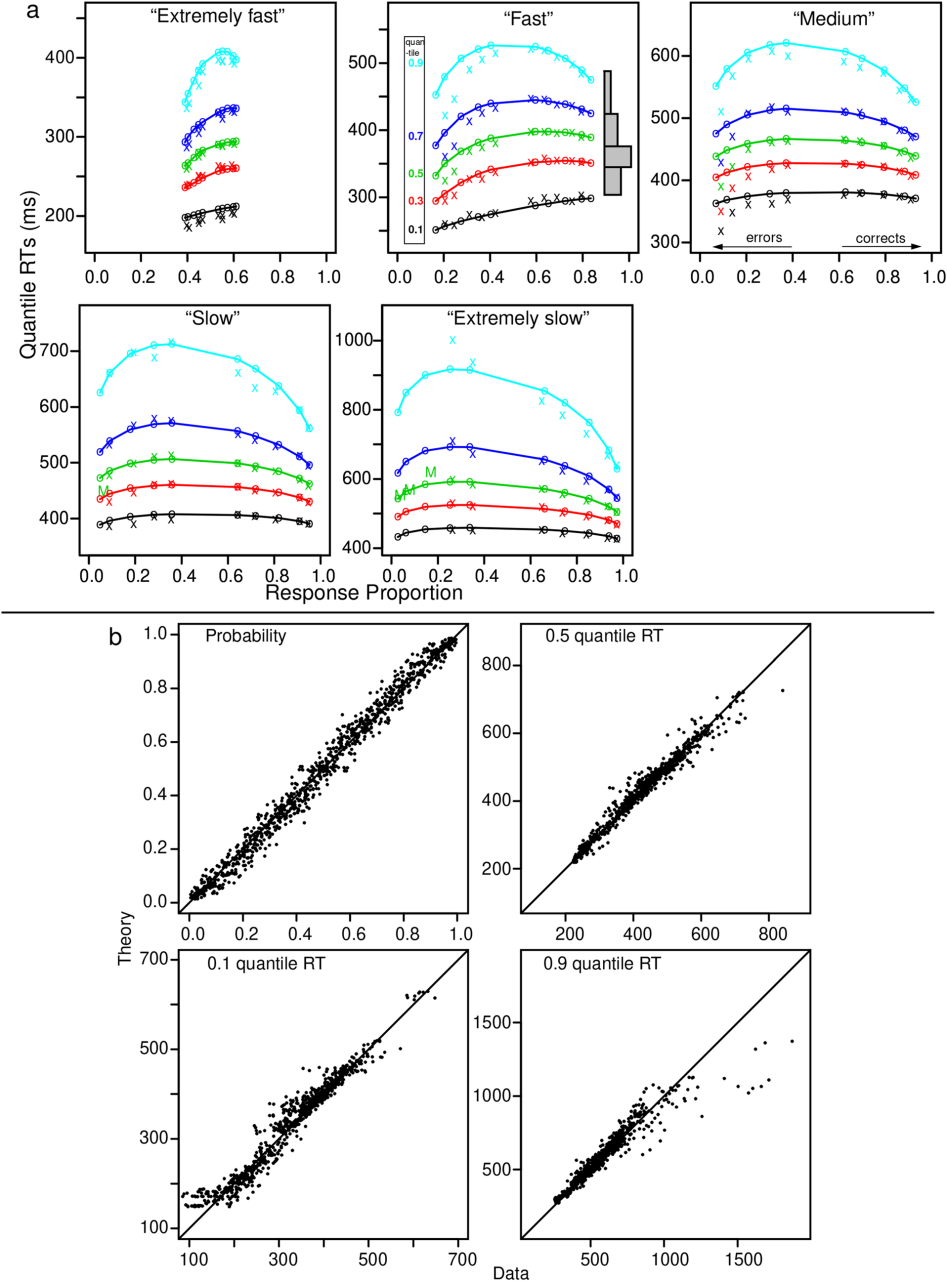
(**a**) Quantile probability plots for the mixture model with data and model predictions averaged over subjects in the same way. The x’s are the data and the o’s are the predictions joined by the lines. The five lines stacked vertically above each other are the values predicted by the diffusion model for the 0.1, 0.3, 0.5, 0.7, and 0.9 quantile RTs as a function of response proportion for the conditions of the experiments, see the top middle panel inset. Equal-area rectangles drawn between the quantiles are shown on the right side of the top middle panel (to show how RT distributions could be constructed). The M’s in the bottom two plots show the median RT because some subjects did not have enough error responses to compute quantiles. (**b**) Plots of accuracy, the 0.1, 0.5 (median), and 0.9 quantile correct response times (RTs) for every subject and every condition. For the quantiles, only values are presented from conditions with over 15 observations.

We show the fits of the model to the data in several ways. First, Fig. 2a shows quantile probability plots for each speed-accuracy condition. The 0.1, 0.3, 0.5, 0.7, and 0.9 quantile RTs are plotted vertically above the proportion of responses for each contrast condition^9,21^. The data are the x’s and the model predictions are the o’s. These plots provide information about how accuracy and the shapes of RT distributions change across the contrast conditions. The shapes can be seen by drawing equal-area rectangles between the quantile RTs, as shown in the top middle panel of Fig. 2a. The 0.1 quantile represents the leading edge of the distribution, the 0.9 quantile represents the tail of the distribution, and the median (0.5 quantile) is the middle row. The change in mean RTs across conditions is mainly a spread in the distributions.

There are two misses between predictions and data. The first is a miss between predictions and data for error RT quantiles in the medium speed-stress condition for the highest-accuracy stimulus contrast condition. The model misses these extreme errors likely because there are low numbers of these errors and so they receive little weight in the model fitting. The second miss is for the 0.1 quantile RTs for the highest speed-stress conditions. The empirical 0.1 quantile RTs are lower than the predictions in Fig. 2b.

The second way we display fits of the model to data is shown in Fig. 2b. Accuracy and the 0.1, 0.5, and 0.9 quantile RTs for predictions and data are plotted against each other for every subject and every condition. These include error RTs for conditions with more than 15 observations. These show almost no systematic deviations between predictions and data.

**Figure 3:**
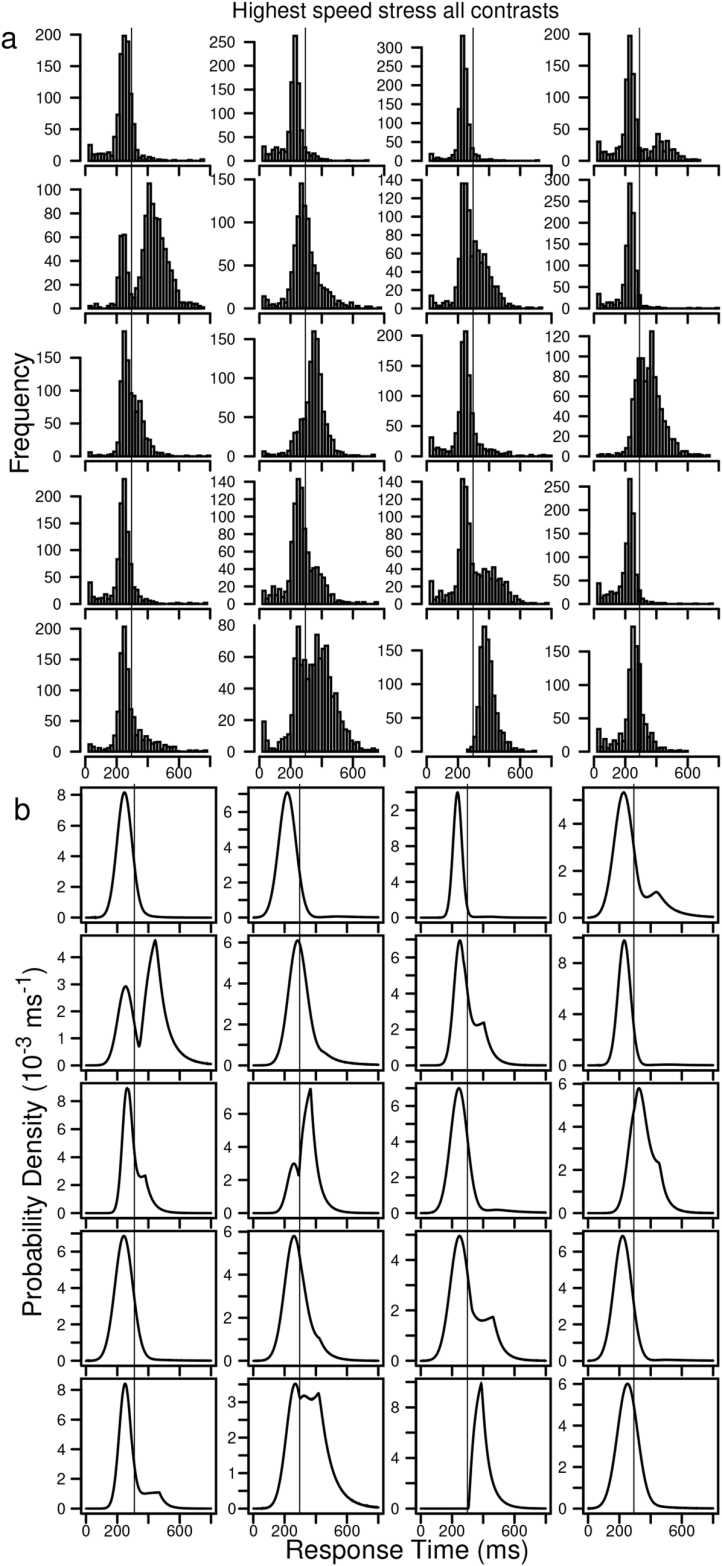
(**a**) Empirical correct RT distributions for the highest speed-stress conditions (collapsed over the levels of stimulus contrast) for each of the 20 individual subjects. (**b**) The same plots for predictions generated from fits of the mixture model to the data. The thin vertical lines at 300 ms provide an approximate way of separating fast guesses (those below 300 ms) from stimulus-based responses.

Figure 3a and 3b show the empirical and predicted RT distributions (respectively) for all the contrast conditions collapsed for the highest speed-stress condition for each individual subject. The thin vertical lines are at 300 ms and if a distribution lies mainly below this, the responses are mainly fast guesses (see Fig. 1a). There are two main patterns of results. Some subjects produce histograms with mostly fast guesses. Other subjects show a pattern with bimodal RT distributions with the two peaks corresponding to fast guesses and to stimulus-based decisions. For all the subjects, the predicted RT distributions qualitatively match the empirical distributions.

Figure 3a shows why the second miss in Fig. 2a occurs. For all the subjects except one, the fastest responses had RTs of exactly 31 ms showing that these subjects were hitting the response key in anticipation of the stimulus (31 ms is too fast for a response to the onset of a stimulus). These extremely fast responses in the left tail produce 0.1 quantile RTs that are lower than a normal distribution can accommodate.

The G-square statistic that is minimized in fitting the model to data is asymptotically distributed as chi-square and so the values from fits to data can be compared with the critical value of chi-square. For 450 degrees of freedom (475–25), the critical value at the 0.95 level is 500.5. The mean value from the fits to the data is 654.4 which is larger than the critical value. However, examination of other diffusion model fits with this many data points per subject (close to 5000 observations per subject) has found that a value between one and two times the critical value represents quite good fits^22^. This is because the G-square statistic is a function of the number of observations and small deviations between theory and data are magnified as the number of observations increases, leading to significant values.

Above, we noted that the normal distribution for the guessing process might be replaced by a more principled distribution. For example, Smith^18^ has a model for simple RT that was composed of a mixture of a rapid stimulus detection process and a slower level detection process. This detection model might be used here instead of the normal, but like the normal, it would miss the leading edge of the RT distributions when there are RTs too short to be due to detection of the stimulus onset (e.g., 31 ms, Fig. 3a).

Rafiei and Rahnev used a t-test to examine whether responses when speed-stress was high had accuracy above chance and so could not be explained by detection of the stimulus onset. Accuracy was above chance so they concluded that accuracy in this highest speed-stress condition was not based completely on detection of the stimulus. However, Fig. 1a shows that responses under 300 ms were at chance. We performed a t-test using mean accuracy values for each subject for all responses for the highest speed-stress condition collapsed over stimulus contrast conditions and for responses with times under 300 ms from those conditions. For all responses, results replicated Rafiei and Rahnev with t(19) = 3.73, p = 0.0014, Cohen’s d = 0.835 (mean accuracy values were 0.566 and 0.434). But for responses with RTs less than 300 ms, accuracy was not significantly different from chance, t(19) = 1.07, p = 0.30, Cohen’s d = 0.240 (mean accuracy values were 0.517 and 0.483). Thus, fast responses (with RTs less than 300 ms) can be considered detection responses in Rafiei and Rahnev’s terms.

Rafiei and Rahnev used three patterns in their data as benchmarks against which to test the diffusion model. None of these refuted the mixture model.

**Figure 4:**
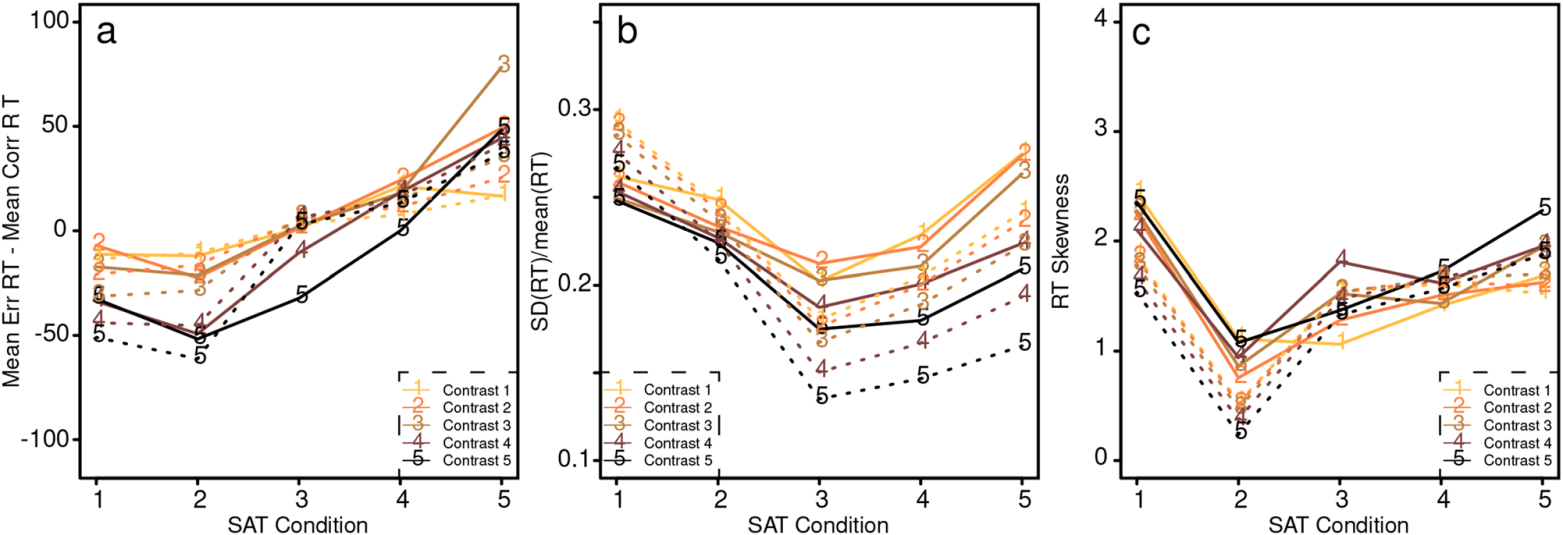
Plots of (**a**) mean error RT minus mean correct RT, (**b**) the SD in RT divided by the mean, and (**c**) RT skewness, each for the 5 speed-accuracy (SAT) conditions. The solid lines are the data and the dotted lines are the theoretical predictions. These are the qualitative “benchmark” analyses that Rafiei and Rahnev claim the diffusion model cannot capture.

The first benchmark was that errors were faster than correct responses for the higher speed-stress conditions but slower than correct responses for the lowest speed-stress condition (Fig. 4a). The mixture model accounts for this because for the higher speed-stress conditions, more of the errors were fast guesses and the number of these dominated errors from the diffusion process, making errors faster than correct responses. As speed-stress decreased, the number of fast guesses declined to near zero and so across-trial variability in drift rate produced errors slower than correct responses (e.g.^9^). The mixture model gives good, quantitative matches with this pattern of data (Fig. 4a).

The second benchmark was how the ratio between the SD and mean of RT distributions changed as a function of speed-stress. The ratio was large for higher speed-stress conditions, then fell for the moderate speed-stress condition, and then rose again for lower speed-stress conditions (Fig. 4b). The mixture model accounts for this pattern for much the same reason as for the first benchmark. The spread of RTs (SD) was larger for the conditions that contained many fast guesses (higher speed-stress), narrower as the number of fast guesses decreased (moderate speed-stress). For the lower speed-stress conditions, the number of fast guesses approached zero but boundary separation increased which led to an increase in the spread of RTs and hence the SD relative to the mean. The mixture model fit this U-shaped function well (Fig. 4b).

The third benchmark was that the skewness of the RT distributions was large at the highest speed-stress condition, then decreased for the next highest speed-stress condition, then increased with further reduced speed-stress. In the mixture model, for the highest speed-stress condition, the proportion of fast guesses was about 0.75 and the mixture of these and responses from the diffusion process produced right skewed RT distributions with large skewness (e.g., Fig. 1a). At the second highest speed-stress condition, the proportion of fast guesses is lower which reduces skewness. As speed-stress is reduced further and the proportion of fast guesses approaches zero, skewness rises again as RT distributions spread with increasing boundary separation. This produces the U shaped functions in Fig. 4c.

Rafiei and Rahnev argued that the diffusion model was incapable of even qualitatively accounting for the patterns of results in these three benchmarks. However, the results in Fig. 4 show the mixture model produces results that match the data.

Although the patterns of results in Figs. 4b and 4c match the qualitative patterns of results, there are problems with use of these measures. First, Ratcliff^12^ presented an analysis of moments of RT distributions that showed that both the SD and skewness have high variability associated with them. He showed that cutoffs of 2 versus 5 s in real data with mean RT around 700 ms can change the SD by 30% and skewness by a factor of 2. Second, these measures are extremely sensitive to outliers^12^. Third, Ratcliff^11^ showed that the skewness measure does not reflect intuitions about skew in distributions (the spread in the tail relative to the rise in the leading edge). Better measures are quartile skewness or Pearson’s skewness measures. Ratcliff found that the skewness measure did not correlate highly with quartile skewness or Pearson’s skewness measures (which correlated with each other) using Monte Carlo simulations. Fourth, there is a question as to how some of the quantities might be calculated. One could compute an overall SD over all the data for a subject or compute a SD for each subject in each experimental condition and average those.

Because the measures that Rafiei and Rahnev propose as benchmarks are quite variable for any individual with potentially large effects of outliers, we argue that this makes them less useful as quantitative benchmarks. Also, because the unusual patterns of results occur because of extensive fast guessing, we argue that this makes them less useful as qualitative benchmarks. But most importantly, the mixture model produces the qualitative U-shaped patterns of results that Rafiei and Rahnev argued showed failure of the diffusion model.

### Ethics statement

No human subjects were involved or enrolled for this article. All data were from a previously published article^1^.

## Discussion

In the research here, we fit the mixture model to the data from Rafiei and Rahnev^1^. The model assumes a simple normal distribution for fast guesses and a diffusion process for discriminative responses. It fit all aspects of the data for individual subjects including the bimodal RT distributions for some subjects, correct and error RT distributions, and accuracy as a function of both stimulus contrast and speed-accuracy conditions. This was done with considerable model constraint: There was the same guessing proportion for all stimulus contrast conditions for each speed-accuracy condition (as well as different boundary separation and nondecision times for each speed-accuracy condition), and there were different drift rates for each stimulus contrast condition but these were the same over all the speed-accuracy conditions. All in all, there were 475 degrees of freedom in the data explained by 25 model parameters.

Rafiei and Rahnev claimed that the standard diffusion model could not account for their data, but their data are contaminated by responses so fast that they could not have come from decision processes that a diffusion model was designed to represent. The history of research with RT measures has made it extremely clear that mixture models are the most appropriate for data that include fast guesses^3–5^. This means that we should expect a mixture model to account for their data and the analyses in this article show that it does, providing a good quantitative and qualitative account of their data.

Rafiei and Rahnev argued that the diffusion model fails to show selective influence, but in the fits presented here, selective influence was found. Stimulus contrast affected only drift rate and the speed-stress manipulation affected boundary separation, nondecision time, and the proportion of fast guesses but it did not affect drift rates. Early in development of the diffusion model, speed-accuracy manipulations were assumed to affect only boundary separation^23^, but later they were found to affect both boundary separation and nondecision time^24^. If speed is stressed to a high degree and fast guessing is avoided, subjects may encode stimulus information to a lesser degree and drift rates may be lower^25^. In Rafiei and Rahnev’s experiment, the mixture model provided excellent fits with selective influence.

Rafiei and Rahnev admitted that there might be other model-based approaches that could account for their data, but they did not evaluate them. Here we implemented a mixture model and found it provided a compact explanation of all the data. The conclusion is that the diffusion model can easily and accurately handle speed-accuracy stress with the addition of a fast-guess process, a process that has been part of research on RTs since the 1960’s.

## Data Availability

Code is available from the first author on request.

## References

[1] Rafiei F, Rahnev D (2021). Qualitative speed-accuracy tradeoff effects that cannot be explained by the diffusion model under the selective influence assumption. Sci. Rep..

[2] Ollman RT (1966). Fast guesses in choice reaction time. Psychon. Sci..

[3] Ollman RT, Billington MJ (1972). The deadline model for simple reaction times. Cognitive Psychol..

[4] Yellott JI (1971). Correction for guessing and the speed-accuracy tradeoff in choice reaction time. J. Math. Psychol..

[5] Luce RD (1986). Response Times.

[6] Blough DS (1978). Reaction times of pigeons on a wavelength discrimination task. J. Exp. Anal. Behav..

[7] Swensson RG (1972). The elusive tradeoff: Speed versus accuracy in visual discrimination tasks. Percept. Psychophys..

[8] Ratcliff R (1978). A theory of memory retrieval. Psychol. Rev..

[9] Ratcliff R, McKoon G (2008). The diffusion decision model: Theory and data for two-choice decision tasks. Neural Comput..

[10] Ratcliff R (2013). Parameter variability and distributional assumptions in the diffusion model. Psychol. Rev..

[11] Ratcliff R (1993). Methods for dealing with reaction time outliers. Psychol. Bull..

[12] Ratcliff R (1979). Group reaction time distributions and an analysis of distribution statistics. Psychol. Bull..

[13] Ratcliff R, Tuerlinckx F (2002). Estimating the parameters of the diffusion model: Approaches to dealing with contaminant reaction times and parameter variability. Psychon. Bull. Rev..

[14] Vandekerckhove J, Tuerlinckx F (2008). Diffusion model analysis with MATLAB: A DMAT primer. Behav. Res. Methods.

[15] Simen P, Contreras D, Buck C, Hu P, Holmes P, Cohen JD (2009). Reward rate optimization in two-alternative decision making: Empirical tests of theoretical predictions. J. Exp. Psychol. Human.

[16] Ratcliff, R. & Hendrickson, A. T. Do data from Mechanical Turk subjects repliate accuracy, response time, and diffusion modeling results? *Behav. Res. Methods* (2021). 10.3758/s13428-021-01573-x 10.3758/s13428-021-01573-x PMC8641698 33825128

[17] Smith, P. L. & Ratcliff, R. Modeling evidence accumulation decision processes using integral equations: Urgency gating and collapsing boundaries. *Psychol. Rev.* (in press). 10.1037/rev0000301 PMC8857294 34410765

[18] Smith PL (1995). Psychophysically principled models of visual simple reaction time. Psychol. Rev..

[19] Ratcliff R, Van Dongen HPA (2011). A diffusion model for one-choice reaction time tasks and the cognitive effects of sleep deprivation. Proc. Natl. Acad. Sci. USA.

[20] Ratcliff R, Childers R (2015). Individual differences and fitting methods for the two-choice diffusion model. Decision.

[21] Ratcliff R, Smith PL, McKoon G (2015). Modeling regularities in response time and accuracy data with the diffusion model. Curr. Dir. Psychol. Sci..

[22] Ratcliff R, Thapar A, Gomez P, McKoon G (2004). A diffusion model analysis of the effects of aging in the lexical-decision task. Psychol. Aging.

[23] Ratcliff R, Rouder JN (1998). Modeling response times for two-choice decisions. Psychol. Sci..

[24] Ratcliff R (2006). Modeling response signal and response time data. Cognitive Psychol..

[25] Starns JJ, Ratcliff R, McKoon G (2012). Evaluating the unequal-variability and dual-process explanations of zROC slopes with response time data and the diffusion model. Cogn. Psychol..

